# Supplementary material for: Mitochondria are positioned at dendritic branch induction sites, a process requiring rhotekin2 and syndapin I
Source: Nat Commun. 2025 Mar 10;16:2353. doi: 10.1038/s41467-025-57399-0 (PMC11893792; doi:10.1038/s41467-025-57399-0)
Supplement: Supplementary file 1 — Supplementary Information [file 41467_2025_57399_MOESM1_ESM.pdf]

Supplementary Data

Mitochondria are positioned at dendritic branch induction sites, a process requiring rhotekin2 and syndapin I

Jessica Tröger[1], Regina Dahlhaus[1§], Anne Bayrhammer[1], Dennis Koch[1], Michael M. Kessels[1*], Britta Qualmann[1*]

[1] Institute of Biochemistry I, Jena University Hospital - Friedrich Schiller University Jena, Nonnenplan 2-4, 07743 Jena, Germany

[§] present address: Research Division for Neurodegenerative Diseases, Faculty of Medicine/Dentistry, Danube Private University, Steiner Landstraße 124, 3500 Krems-Stein, Austria

* Correspondence

Britta.Qualmann@med.uni-jena.de and Michael.Kessels@med.uni-jena.de

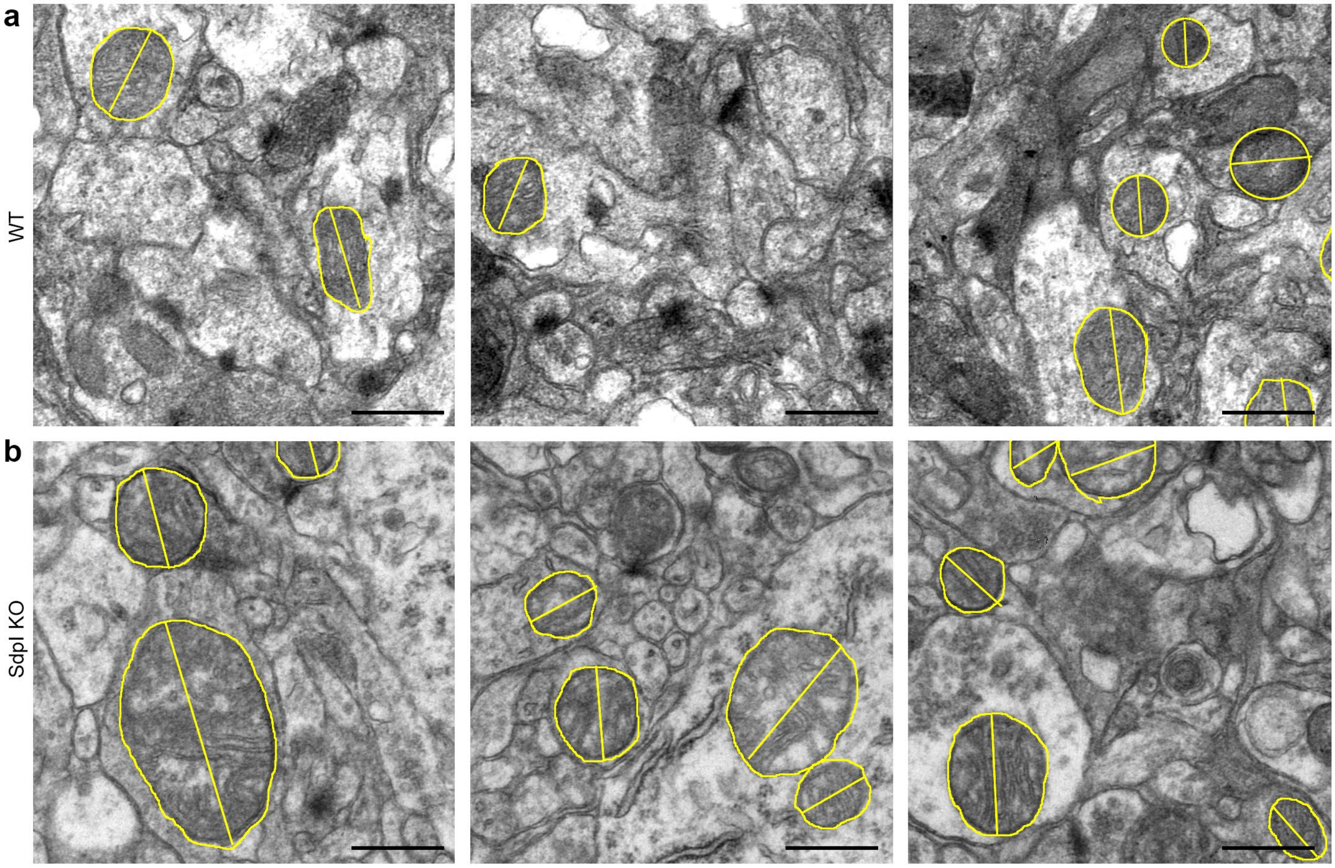

Supplementary Fig. 1. *Syndapin I* KO leads to larger mitochondria

(**a,b**) Details of transmission electron microscopy images of brain sections of WT (**a**) and *syndapin I* KO (SdpI KO) mice (**b**) (age, approx. 20 weeks) showing mitochondria in neurons of the hippocampal CA3 region at higher magnification than in corresponding main figure (**Fig. 1a,b)** and with marks showing mitochondrial area and length determinations (yellow lines; not completely shown in all cases due to the fact that the images shown are details of larger images used for quantitative analyses). Bars, 500 nm.

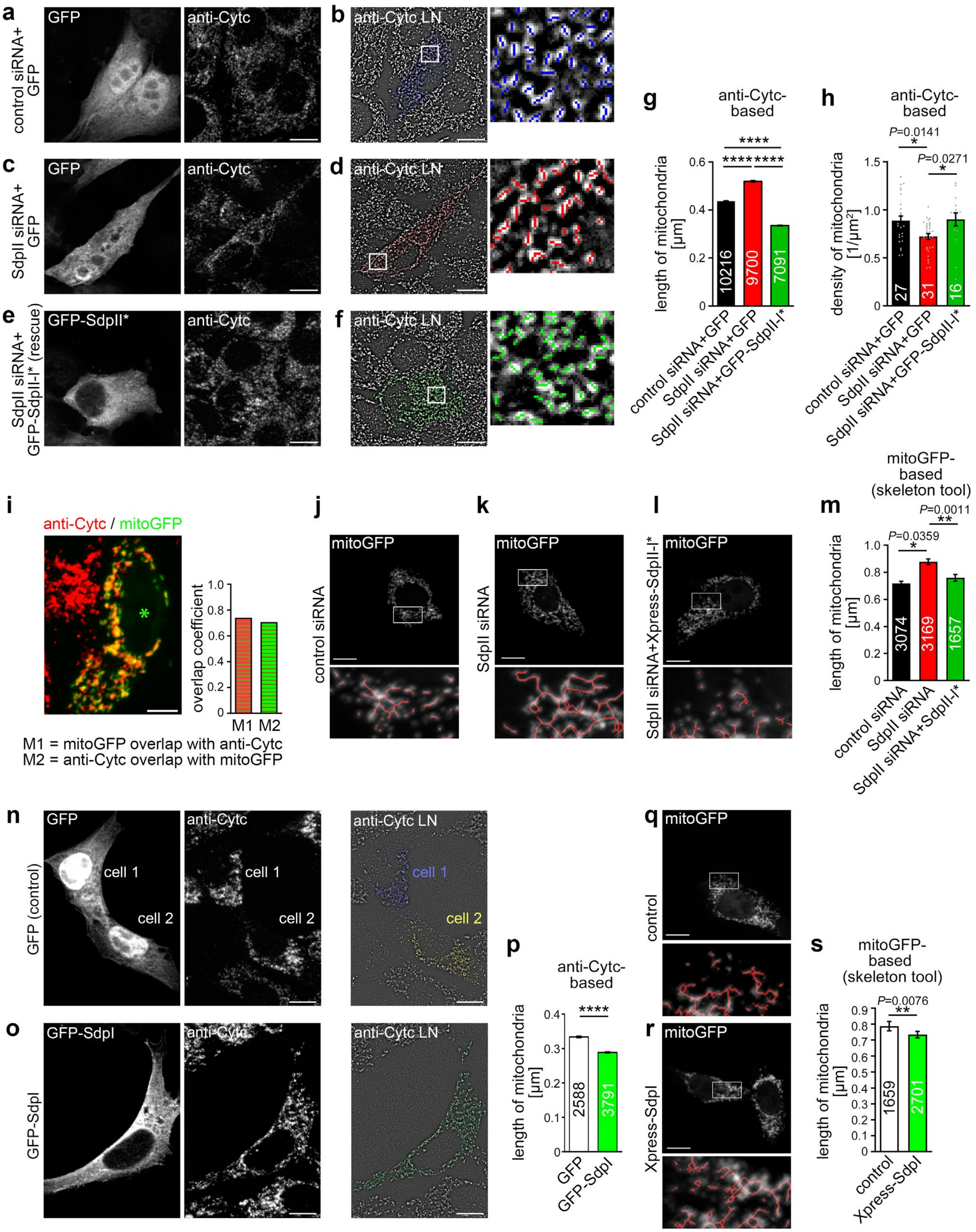
a
control siRNA+ GFP
GFP
anti-Cytc
b
anti-Cytc LN
c
SdpII siRNA+ GFP
d
e
SdpII siRNA+ GFP-SdpII-I* (rescue)
GFP-SdpII*
f
g
anti-Cytc-based
length of mitochondria [µm]
10216
9700
7091
control siRNA+GFP
SdpII siRNA+GFP
SdpII siRNA+GFP-SdpII-I*
h
P=0.0141
P=0.0271
density of mitochondria [1/µm²]
27
31
16
i
anti-Cytc / mitoGFP
overlap coefficient
M1 M2
M1 = mitoGFP overlap with anti-Cytc
M2 = anti-Cytc overlap with mitoGFP
j
control siRNA
mitoGFP
k
SdpII siRNA
l
SdpII siRNA+Xpress-SdpII-I*
m
mitoGFP-based (skeleton tool)
P=0.0359
P=0.0011
3074
3169
1657
control siRNA
SdpII siRNA
SdpII siRNA+SdpII-I*
n
GFP (control)
cell 1
cell 2
o
GFP-SdpI
p
2588
3791
GFP
q
control
r
Xpress-SdpI
s
P=0.0076
1659
2701
control

Supplementary Fig. 2. Syndapin II loss-of-function leads to larger and fewer mitochondria and syndapin I gain-of-function correspondingly results in shortened mitochondria

(**a-f**) Sum intensity projections of ApoTome images of control siRNA-treated NIH3T3 cells (**a,b**), acute knockdown of syndapin II (SdpII siRNA) (**c,d**) and SdpII siRNA cells reexpressing RNAi-resistant GFP-SdpII-l* (SdpII siRNA+GFP-SdpII-l*; rescue) (**e,f**) stained with antibodies against the mitochondrial marker cytochrome c (Cytc) (**a,c,e**) and mitochondrial representations using the *local normalization* (LN) plug-in of ImageJ to filter the anti-Cytc fluorescence signals for discrimination of individual mitochondria (Cytc LN) (**b,d,f**), respectively. Colored lines (particularly visible in the enlargements of the boxed image boxed areas; **b,d,f**, right panels) show mitochondrial length measurements. (**g,h**) Quantitative analyses showing that RNAi against the respective syndapin isoform (SdpII) resulted in significantly elongated mitochondria present at a lower density. Rescue experiments with an RNAi-insensitive syndapin II (SdpII-l*) demonstrate the specificity of these phenotypes. (**i**) Colocalization experiments and Manders M1 and M2 overlap coefficient determinations of mitochondria immunostained for endogenous Cytc and of mitochondria filled by overexpression of mitoGFP (transfected cell marked by asterisk). Note that both anti-Cytc and mitoGFP overexpression visualize mitochondria equally well and show similar overlap coefficients. (**j-m**) Additional mitochondrial length determinations similar to those shown in **a-g** based on mitoGFP overexpression. Magnifications shown below represent boxed areas in the main figures panels and include the tracings of the skeleton software tool (red lines). Note that the syndapin II RNAi phenotype is fully and consistently reproduced irrespective of method used for mitochondrial length determinations. (**n,o**) Anti-Cytc immunostained NIH3T3 cells overexpressing GFP (control) (**n**) and GFP-syndapin I (**o**), respectively. Right panels show the anti-Cytc immunofluorescence signal filtered using the *local normalization* plug-in in ImageJ (Cytc LN) enabling discriminations and length determinations of individual mitochondria. Measurement lines are documented in blue (transfected cell 1; **n**) and yellow

(transfected cell 2; **n**) and in green (transfected cell in **o**). (**p**) Determinations of mitochondrial length in NIH3T3 cells overexpressing GFP and GFP-SdpI, respectively. (**q-s**) Related evaluations of mitochondrial length based on the use of overexpressed mitoGFP and the skeleton tool. Note that also the syndapin I gain-of-function phenotype is reproduced irrespective of method used. Bars, 10 µm. Data, bar plots (mean±SEM) in part (**h**) additionally overlaid with individual data points. Control siRNA+GFP, *n*=10216; SdpII siRNA+GFP, n=9700 and SdpII siRNA+GFP-SdpII-l*, *n*=7091 mitochondria (**g**). Control, siRNA+GFP, *n*=27, SdpII siRNA+GFP, *n*=31 and SdpII siRNA+GFP-SdpII-l*, *n*=16 cells (**h**). *n*=3074 (control siRNA), *n*=3169 (SdpII siRNA) and *n*=1657 (SdpII siRNA+Xpress-SdpII-l*) mitochondria for corresponding mitoGFP/skeleton tool-based analyses (**m**). GFP, *n*=2588; GFP-SdpI, *n*=3791 mitochondria (**p**) as well as *n*=1659 (control) and *n*=2701 (Xpress-SdpI) (**s**). Kruskal-Wallis/Dunn´s post-test (**g,m**), One-way ANOVA/Tukey´s posttest (**h**) and Mann-Whitney (**p,s**), respectively.* $P<0.05$; ** $P<0.01$; **** $P<0.0001$ (for exact P values (≥0.0001) see figure).

a

```
mouse rhotekin1     MFSRNHRSRITVARGSALEMEFKRGRFRLSFFSESPEDTELQRKLDHEIRMRDGACKLLA  60
mouse rhotekin2     -----------------MEGQLLRG-------LAAQQDCSIREKIDLEIRMREGIWKLLS  36
                                     :* :: **         : :* .::.*:* *****:*  ***:

mouse rhotekin1     ACSQREQALEATKSLLVCNSRILSYMGELQRRKEAQVLEKTGRRPS-DSVQPAQHSPCRG  119
mouse rhotekin2     LSTKKDQVLHAVKNLMVCSARIQAYTAELQKSKEEIANQTGARLVLDSSSENKEGESCRG  96
                     .::::*.*.*.*.*:**.:** :* .***: **  . :. .*    .* :  : . ***

mouse rhotekin1     RVCISDLRIPLMWKDTEYFKNKGDLHRWAVFLLLQIGEQIQDTEMVLVDRTLTDISFQNN  179
mouse rhotekin2     KIALSDIRIPLMWKDSDHFSNKECTQRFAIFCLFRMGAQVFDTDMVIVDQTVTDICFENV  156
                    ::.:**:********:::*.**   :*:*:* *:::* *: **:**:**:*:***.*:*

mouse rhotekin1     VLFAEAEPDFELRLELYGACVEEEGALAGAPKRLATKLSSSLGRSSGKRVRASLDSAGAS  239
mouse rhotekin2     TIFNEAGPDFQIKIEVYS-CSAEESSLTNTPRKLAKKLKTSISKATGRKISAALQEESP-  214
                    .:* ** ***::::*:*. *  **.:*:.:*::**.**.:*:.:::*::: *:*:. .

mouse rhotekin1     GNSPVLLPTPAVGGPRFHLLAHTTLTLEEVQDGFRTHDLTLTSHEENPAWLPLYGSVCCR  299
mouse rhotekin2     ---EACLLAGSVAGAKYHLLAHTTLTLENAGDCFKTHNLSVHGDEECSFWLPLYGNVCCR  271
                        . * : :*.* ::***********:. * *:**:*:: ..**   ******.****

mouse rhotekin1     LAAQPLCMIQPTASGALRVQQAGELQNG-TLVHGVLKGTNLFCYWRSEDADTGQEPLFTI  358
mouse rhotekin2     LVAQPACMAADAFAGFLNEQQTGKGLVGWRRLYCALRGGKLRCFYGPEEIEAKVEPALVV  331
                    *.*** **   : :* *. **:*:   *   :: .*:* :* *::  *: ::  ** :.:

mouse rhotekin1     VINKETRVRAGELEQAPEWPFTLSISNKYGDDEVTNTLQLESREALQNWMEALWQLFFDM  418
mouse rhotekin2     PIDKETRIQAVEKDSKKMHCFS--VLSTAAGRAVSHIFAADSLADFQEWMGAFRQHFFDL  389
                     *:****::* * :.     *:  : .. ..  *:: :  :*   :*:** *: * ***:

mouse rhotekin1     SQWRHCCDEVMKIETPAPRKPPQALAKQG-SLYHEMAIEPLDDIAAVTDILAQREGT---  474
mouse rhotekin2     SQWKHCCEELMRIEIMSPRKPPLFLAKEATSVYYDMSIDSPVKLESVTDIIQKKIGETNR  449
                    ***:***:*:*:**  :*****  ***:. *:*::*:*:   .: :****: :: *

mouse rhotekin1     -------RLEPSPPWLAMFTDQPALPSSCS-------PASVAPVPTWMQPLPWGRPRTFS  520
mouse rhotekin2     QFLIGRDDQSAAPPWAAVFDGNHEMVIEKKVLSPIGEPAPDGKRKKRRAPLPPTDQPPFC  509
                             . :*** *:* .:  :  . .       **  .   .   ***      *.

mouse rhotekin1     LDAAPAD-HS------LGP-SRSVAPLPPQRSPK--------------------------  546
mouse rhotekin2     IKTQGRANQSKDSATQAGVSGASSSPSDPRLSPPTHHLQKPVAAPRKLLPARKNSSADIG  569
                    :.:     :*       *  . * :*  *: **

mouse rhotekin1     -----------------SRGFYSKSQLGPWLQSPV   564
mouse rhotekin2     HTDTKTSLDAKPVPVPRQKSIRDILDPRSWLQAQV   604
                                     .:.: .  :   ***: *
```

b

```
mouse rhotekin2`    MEGQLLRGLAAQQDCSIREKIDLEIRMREGIWKLLSLSTKKDQVLHAVKNLMVCSARIQA   60
mouse rhotekin2     MEGQLLRGLAAQQDCSIREKIDLEIRMREGIWKLLSLSTKKDQVLHAVKNLMVCSARIQA   60

mouse rhotekin2`    YTAELQKSKEEIANQTGAR---DSSSENKEGESCRGKIALSDIRIPLMWKDSDHFSNKEC   117
mouse rhotekin2     YTAELQKSKEEIANQTGARLVLDSSSENKEGESCRGKIALSDIRIPLMWKDSDHFSNKEC   120

mouse rhotekin2`    TQRFAIFCLFRMGAQVFDTDMVIVDQTVTDICFENVTIFNEAGPDFQIKIEVYSCSAEES   177
mouse rhotekin2     TQRFAIFCLFRMGAQVFDTDMVIVDQTVTDICFENVTIFNEAGPDFQIKIEVYSCSAEES   180

mouse rhotekin2`    SLTNTPRKLAKKLKTSISKATGRKISAALQEESPEACLLAGSVAGAKYHLLAHTTLTLEN   237
mouse rhotekin2     SLTNTPRKLAKKLKTSISKATGRKISAALQEESPEACLLAGSVAGAKYHLLAHTTLTLEN   240

mouse rhotekin2`    AGDCFKTHNLSVHGDEECSFWLPLYGNVCCRLVAQPACMAADAFAGFLNEQQTGKGLVGW   297
mouse rhotekin2     AGDCFKTHNLSVHGDEECSFWLPLYGNVCCRLVAQPACMAADAFAGFLNEQQTGKGLVGW   300

mouse rhotekin2`    RRLYCALRGGKLRCFYGPEEIEAKVEPALVVPIDKETRIQAVEKDSKKMHCFSVLSTAAG   357
mouse rhotekin2     RRLYCALRGGKLRCFYGPEEIEAKVEPALVVPIDKETRIQAVEKDSKKMHCFSVLSTAAG   360

mouse rhotekin2`    RAVSHIFAADSLADFQEWMGAFRQHFFDLSQWKHCCEELMRIEIMSPRKPPLFLAKEATS   417
mouse rhotekin2     RAVSHIFAADSLADFQEWMGAFRQHFFDLSQWKHCCEELMRIEIMSPRKPPLFLAKEATS   420

mouse rhotekin2`    VYYDMSIDSPVKLESVTDIIQKKIGETNGQFLIGRDDQSAAPPWAAVFDGNHEMVIEKKV   477
mouse rhotekin2     VYYDMSIDSPVKLESVTDIIQKKIGETNRQFLIGRDDQSAAPPWAAVFDGNHEMVIEKKV   480

mouse rhotekin2`    LSPTGEPAPDGKRKKRRAPLPPTDQPPFCIKTQGSANQSKDSATQAGVSGASPLPSDPRL   537
mouse rhotekin2     LSPIGEPAPDGKRKKRRAPLPPTDQPPFCIKTQGRANQSKDSATQAGVSGASSSPSDPRL   540

mouse rhotekin2`    LLPTHHLQKPVAAPRKLLPARKNSSADIGHTDTKTSLDAKPVPVPRQKSIRDILDPRSWL   597
mouse rhotekin2     SPPTHHLQKPVAAPRKLLPARKNSSADIGHTDTKTSLDAKPVPVPRQKSIRDILDPRSWL   600

mouse rhotekin2`    QAQV   601
mouse rhotekin2     QAQV   604
```

Supplementary Fig. 3. Alignment of mouse rhotekin1 and rhotekin2 as well as alignment of rhotekin2 isoform 1 and isoform 2 with highlighted motifs and domains

(**a**) Alignment of mouse rhotekin1 (RTKN; NP_001129699.1) and rhotekin2 (RTKN2; NP_001346247.1) demonstrating the extremely limited similarities between both proteins. (**b**) Alignment of murine rhotekin2 variants. Shown are rhotekin2 isoform 1 (termed rhotekin2; version from BL6 mice; NP_001346247.1; 604 aa) and rhotekin2 isoform 2 (termed rhotekin2' in this study). Note that the rhotekin2' version from BALB C mice (EDL_32016.1) contains 7 exchanges (aa marked in red) in comparison to rhotekin2 isoform 2 from BL6 mice (NP_001346248.1). Rhotekin2' clone#541 identified as syndapin I binding partner by Y2H screening represented the BALB C sequence, as the mouse brain library used was derived from BALB C mice. Protein domains are colored as in **Fig. 1**. Purple, REM-1 (Rho effector motif class 1), green, pleckstrin homology (PH) and red, a proline-rich domain (PRD). PxxP (proline-x-x-proline) motifs, i.e. putative interaction sites for SH3 domains, are underlined in dark grey (identified KRAP motif contains the second PxxP motif).

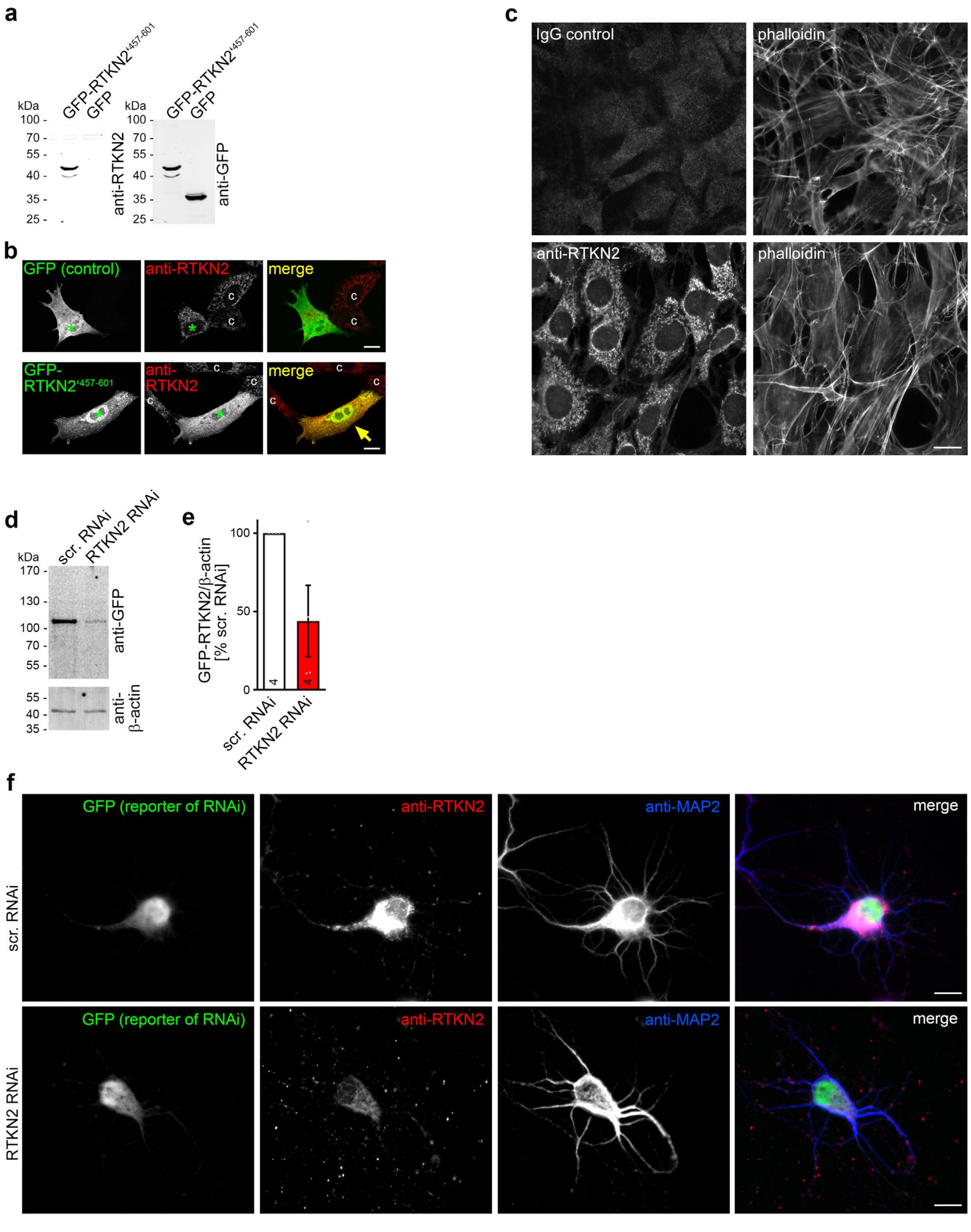
a
kDa
GFP-RTKN2'457-601
GFP
100
70
55
40
35
25
anti-RTKN2
anti-GFP
b
GFP (control)
anti-RTKN2
merge
GFP-RTKN2'457-601
anti-RTKN2
c
IgG control
phalloidin
anti-RTKN2
phalloidin
d
scr. RNAi
RTKN2 RNAi
kDa
170
130
100
70
55
40
35
anti-GFP
anti-β-actin
e
GFP-RTKN2/β-actin [% scr. RNAi]
100
50
0
4
4
scr. RNAi
RTKN2 RNAi
f
GFP (reporter of RNAi)
anti-RTKN2
anti-MAP2
merge
scr. RNAi
RTKN2 RNAi

Supplementary Fig. 4. Characterization of polyclonal guinea pig antibodies raised against rhotekin2 and of the effectiveness of rhotekin2 RNAi

(**a**) Western blot analyses showing the specificity of polyclonal guinea pig antibodies raised and affinity-purified against rhotekin2'$^{457-601}$ in immunoblotting analyses of lysates from HEK293 cells expressing GFP-RTKN2' and GFP, respectively. (**b**) MIPs of immunofluorescence images of transfected NIH3T3 cells showing that the anti-rhotekin2 antibody recognized both endogenous rhotekin2, as highlighted in untransfected control cells (marked by "c"), as well as GFP-RTKN2'$^{457-601}$ (yellow arrow in lower panel) but did not recognize GFP (control; upper panel). Transfected cells are marked by asterisks. (**c**) Immunostainings (MIPs) of endogenous rhotekin2 in NIH3T3 cells. Lower panels show anti-rhotekin2 immunostainings and upper panels show the background obtained by unrelated IgGs (specificity control). As cellular marker, filamentous actin was stained by phalloidin. (**d**) Anti-GFP immunoblot analysis of lysates of HEK293 cells transfected with derivatives of scrambled RNAi (scr. RNAi) and of RTKN2 RNAi vectors additionally encoding for GFP-RTKN2. Anti-β-actin immunoblotting serves as control. (**e**) Quantitative analyses of anti-GFP immunoblotting signals normalized to anti-β-actin signals (scr. RNAi set to 100%) demonstrate a strong reduction of GFP-RTKN2 expression upon RTKN2 RNAi compared to scrambled RNAi (-56%). Data represent mean±SEM (as bar/dot plot); *n*=4 independent assays. (**f**) RTKN2 RNAi- and scr. RNAi-transfected (GFP-reported) developing neurons (DIV7) immunostained against rhotekin2 and the dendritic marker MAP2 (MIPs). The reduced rhotekin2 immunostaining (red in merges) upon RTKN2 RNAi confirms both the specificity of the anti-RTKN2 immunolabeling as well as the effectiveness of the RNAi tool. Bars, 10 µm. All experiments shown as representative images (**a-c,f**) have been done at least twice with similar results.

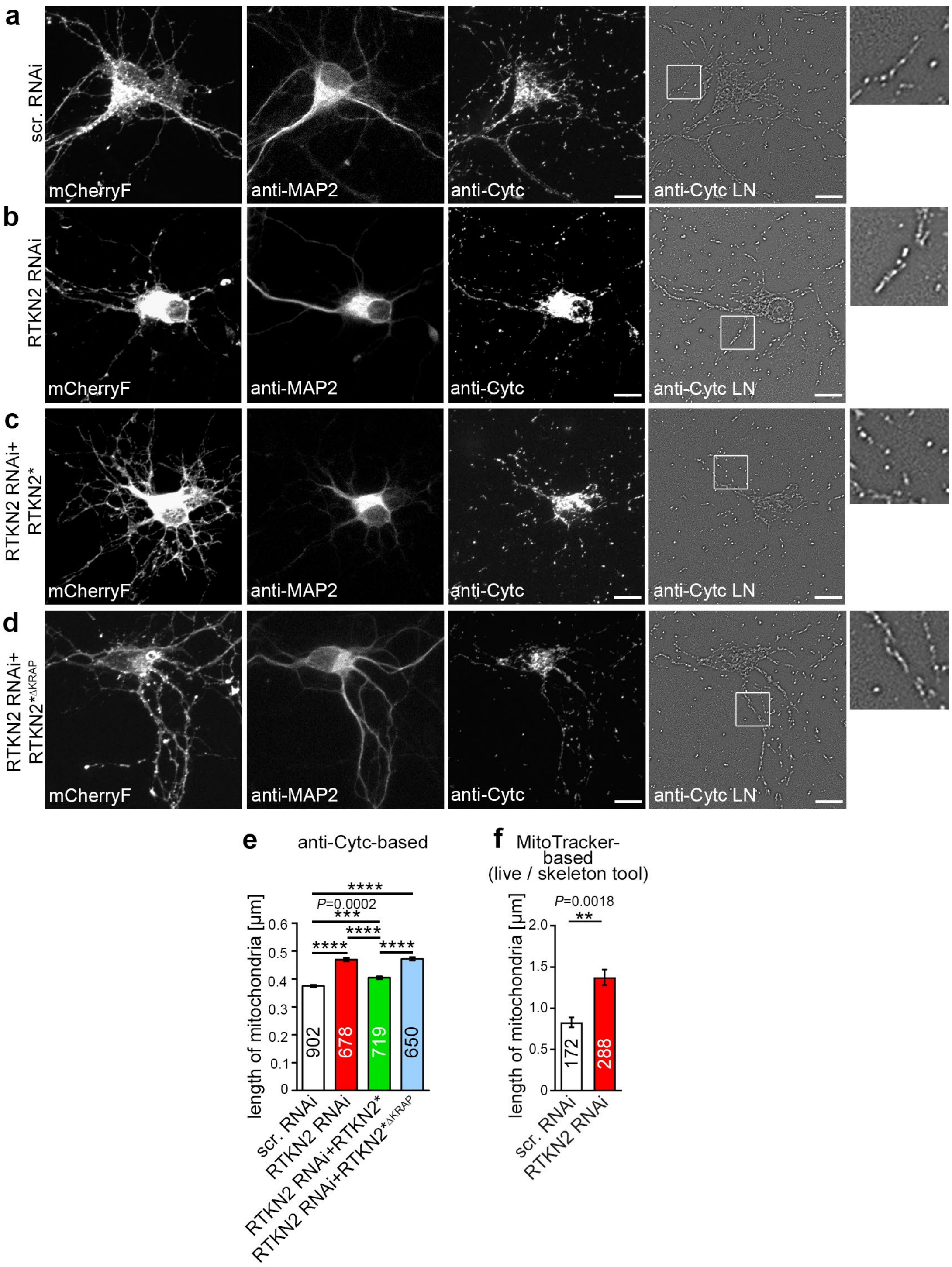

Supplementary Fig. 5. Rhotekin2 shapes mitochondria in the dendritic arbor of hippocampal neurons

(a-d) Analyses of mitochondrial rhotekin2 loss-of-function phenotypes in primary hippocampal neurons transfected at DIV4, fixed 48 h after transfection and immunostained with antibodies against MAP2 and

cytochrome c (Cytc). Right panels show the filtered anti-cytochrome c immunosignal (*local normalization* plug-in of ImageJ; Cytc LN) enabling the discrimination and quantitative evaluation of individual mitochondria. Boxed areas are depicted at higher magnification at the right hand side of the respective image. Shown are example images for scrambled RNAi **(a)**, RTKN2 RNAi **(b)** and combinations of RTKN2 RNAi with coexpression of RNAi-insensitive wild-type rhotekin2 (RTKN2*, **c**) and rhotekin2$^{\Delta KRAP}$ mutant (RTKN2*$^{\Delta KRAP}$, **d**) lacking the syndapin binding motif. Scrambled and RTKN2 RNAi vectors are mCherryF-reported. Bars, 10 µm. **(e)** Quantitative analyses of mean mitochondrial length of all mitochondria in the dendritic compartment. (**f**) Determinations of mitochondrial length in live microscopy data of neurons stained with MitoTracker demonstrating that the length determinations are not hampered by fixation and that the identified rhotekin2 loss-of-function phenotype is not a fixation artifact. Data, mean±SEM visualized as bar plots. (**e**) Scr. RNAi, *n*=902; RTKN2 RNAi, *n*=678; RTKN2 RNAi+RTKN2*, *n*=719 and RTKN2 RNAi+RTKN2*$^{\Delta KRAP}$, *n*=650 mitochondria in 21, 15, 17 and 16 neurons, respectively, from 3 independent assays. (**f**) Scr. RNAi, *n*=172; RTKN2 RNAi, *n*=288 dendritic mitochondria in 13 and 14 neurons from 2 independent assays, respectively. Statistical significances, Kruskal-Wallis/Dunn´s post-test (**e**) and Mann-Whitney (**f**), respectively. ** $P<0.01$, *** $P<0.001$, **** $P<0.0001$. For exact *P* values ≥0.0001 see figure.

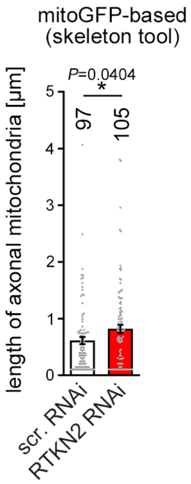

Supplementary Fig. 6. Rhotekin2 deficiency also leads to increased length of axonal mitochondria Quantitative determinations of mitochondrial length in axons of DIV6 hippocampal neurons transfected with either scr. RNAi (control) or RTKN2 RNAi at DIV4. Note that also axonal mitochondria are increased in length upon rhotekin2 deficiency. Data are mean±SEM visualized as bar/dot plot. Scr. RNAi, n=97; RTKN2 RNAi, n=105 axonal mitochondria from 24 (scr. RNAi) and 20 neurons (RTKN2 RNAi), respectively, from two independent preparations of hippocampal cultures evaluated based on mitoGFP tracing and using the skeleton tool of ImageJ. Statistical significance, Mann-Whitney. * P<0.05. For exact P value see figure.

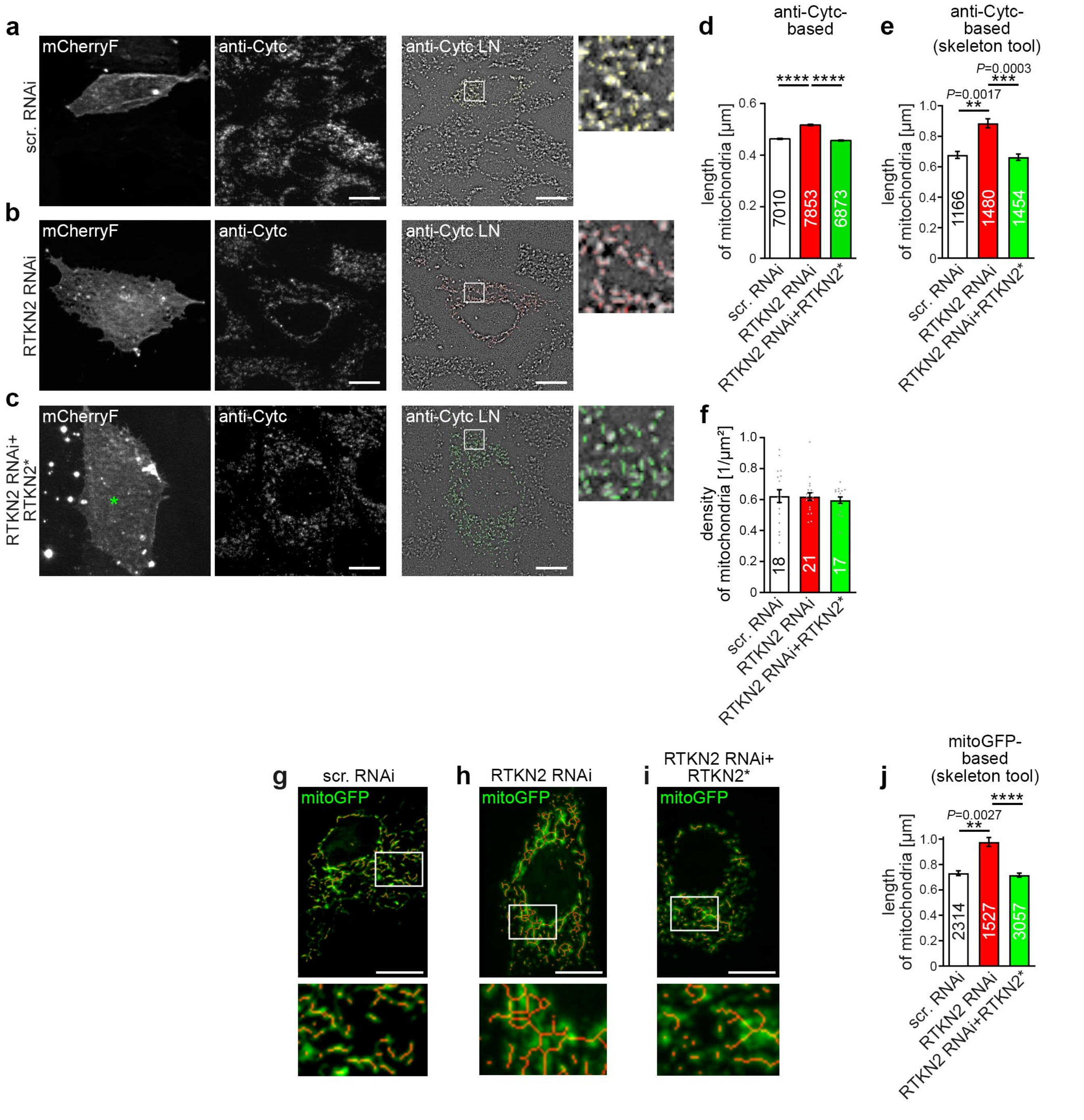

Supplementary Fig. 7. Rhotekin2 loss-of-function effects in NIH3T3 cells mirror the observed defects in primary hippocampal neurons

(**a-f**) Immunofluorescence (**a-c**) and quantitative analyses (**d-f**) of knockdown experiments in NIH3T3 cells. Cells were transfected with control vectors (**a**; mCherryF-reported scrambled RNAi), RTKN2

RNAi without **(b)** and in presence of RNAi-insensitive wild-type rhotekin2 (**c**; RTKN2*, untagged; GFP-reported; bicistronic vector). Left panels, mCherryF reporter signals (additional RTKN2* expression reported by GFP is marked by an asterisk (**c**)); middle panels, anti-cytochrome c (Cytc) immunolabeling; right panels, filtered cytochrome c signal (*local normalization* plug-in ImageJ; Cytc LN). Boxed area in Cytc LN images is shown at higher magnification at the right of the respective image. Individual mitochondria measured are colored (yellow in (**a**), red in (**b**) and green in (**c**)). (**d-f**) Quantitative analyses of mitochondrial length (**d,e**; alternative methods of quantitative evaluation) and density (**f**) show a highly significant increase in mitochondrial length upon RTKN2 RNAi in comparison to control (scrambled RNAi). Coexpression of RTKN2* rescued the phenotype. (**g-j**) Alternative experiments using overexpressed mitoGFP as mitochondrial tracer (green) and the skeleton tool (red lines) for evaluations of mitochondrial length. Note that irrespective of method employed, rhotekin2 deficiency consistently leads to a statistically significant increase in mitochondrial length (compare **d,e** and **j**). Bars, 10 µm. Data, mean±SEM visualized as bar plots (**d,e,j**) and bar/dot plots for examinations with lower *n* numbers (**f**), respectively. (**d**) Scr. RNAi, *n*=7010; RTKN2 RNAi, *n*=7853 and RTKN2 RNAi+RTKN2*, *n*=6873 mitochondria in *n*=18 (scr. RNAi), *n*=21 (RTKN2 RNAi) and *n*=17 (RTKN2 RNAi+RTKN2*) cells (**f**) of 3 independent NIH3T3 cell assays. (**e**) Scr. RNAi, *n*=1166; RTKN2 RNAi, *n*=1480 and RTKN2 RNAi+RTKN2*, *n*=1454 mitochondria in 10 cells each. (**j**) Scr. RNAi, *n*=2314; RTKN2 RNAi, *n*=1527 and RTKN2 RNAi+RTKN2*, *n*=3057 mitochondria in *n*=29 (scr. RNAi) and *n*=30 (RTKN2 RNAi; RTKN2 RNAi+RTKN2*) cells. Statistical significances, Kruskal-Wallis/Dunn´s post-test (**d-f,j**). * $P<0.05$; ** $P<0.01$; *** $P<0.001$; **** $P<0.0001$ (for exact P values (≥0.0001) see figure).

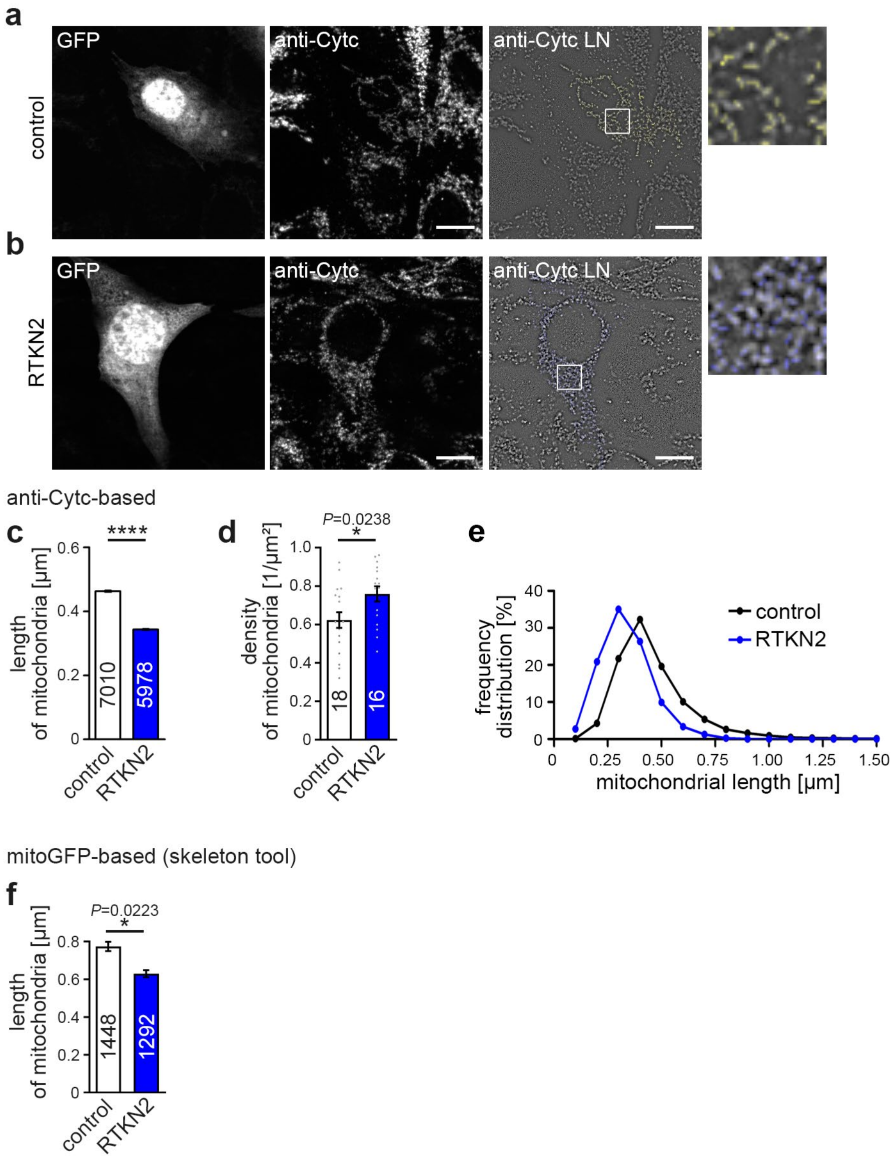

Supplementary Fig. 8. Rhotekin2 gain-of-function studies consistently show the opposite of rhotekin2 loss-of-function, a decline in mitochondrial length

(**a-f**) Rhotekin2 overexpression experiments in NIH3T3 cells and quantitative assessments of mitochondria based on immunolabeling of endogenous cytochrome c (Cytc) (**a-e**) and alternative examinations of mitochondrial length employing overexpression of mitoGFP as mitochondrial tracer and the skeleton tool of ImageJ for evaluation (**f**). (**a,b**) Sum intensity projections (ApoTome) of anti-cytochrome c (Cytc)-stained NIH3T3 cells expressing GFP (**a**) and rhotekin2 (untagged; GFP-reported

by bicistronic vector) (**b**), respectively, and corresponding filtered image by the *local normalization* plug-in of ImageJ (Cytc LN, right panels) enabling the discrimination of individual mitochondria (yellow in **a**; blue in **b**). Boxed area in anti-Cytc LN images is shown at higher magnification right to the respective image. (**c,d**) Quantitative analyses of mitochondrial length (**c**) and density (**d**) based on anti-Cytc immunostaining. (**e**) Frequency distribution of mitochondrial length in GFP (control) or RTKN2 overexpressing NIH3T3 cells revealed a shift to smaller mitochondrial sizes in RTKN2 overexpressing NIH3T3 cells. (**f**) Corroborating quantitative determinations of mitochondrial length using overexpressed mitoGFP and the skeleton tool. Note that similar to the anti-Cytc-based evaluations, also this method consistently shows the rhotekin2 gain-of-function phenotype manifesting in reduced mitochondrial sizes. Bars, 10 µm. Data, mean±SEM visualized as bar plots (**c,f**) and bar/dot plots for examinations with lower *n* numbers (**d**), respectively. (**c**) Control, n=7010 (as scr. RNAi in **Supplementary Fig. 7c**) and RTKN2 (scr. RNAi+RTKN2*), n=5978 mitochondria. (**d**) n=18 (control) (as in **Supplementary Fig 7d**) and n=16 (RTKN2) cells of 3 independent assays. (**f**) Control (scr. RNAi+pIRES), n=1448 and RTKN2 (scr. RNAi+RTKN2*), n=1292 mitochondria in n=20 and n=17 neurons, respectively. Statistical significances, Mann-Whitney (**c,f**); unpaired Student's t-test (**d**). * P<0.05; **** P<0.0001 (for exact P values (≥0.0001) see figure).

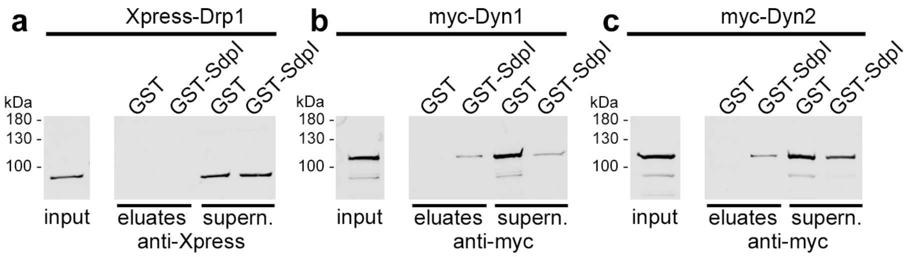

Supplementary Fig. 9. Syndapin I fails to associate with Drp1 but binds to dynamin 2

(**a-c**) Immunoblotting analyses of coprecipitation studies with immobilized GST-SdpI and GST, respectively, and with lysates of HEK293 cells transfected with vectors driving the expression of epitope-tagged versions of dynamin-related protein 1 (Drp1; **a**), dynamin1 (Dyn1; **b**) und dynamin2 (Dyn2;**c**). All experiments shown as representative images (**a-c**) have been done at least twice and showed similar results. White lines between input and coprecipitation samples represent standard lanes omitted. Note that, while Drp1 does not interact with syndapin I (**a**), in addition to dynamin1 (**b**), also dynamin2 associates with syndapin I (**c**).

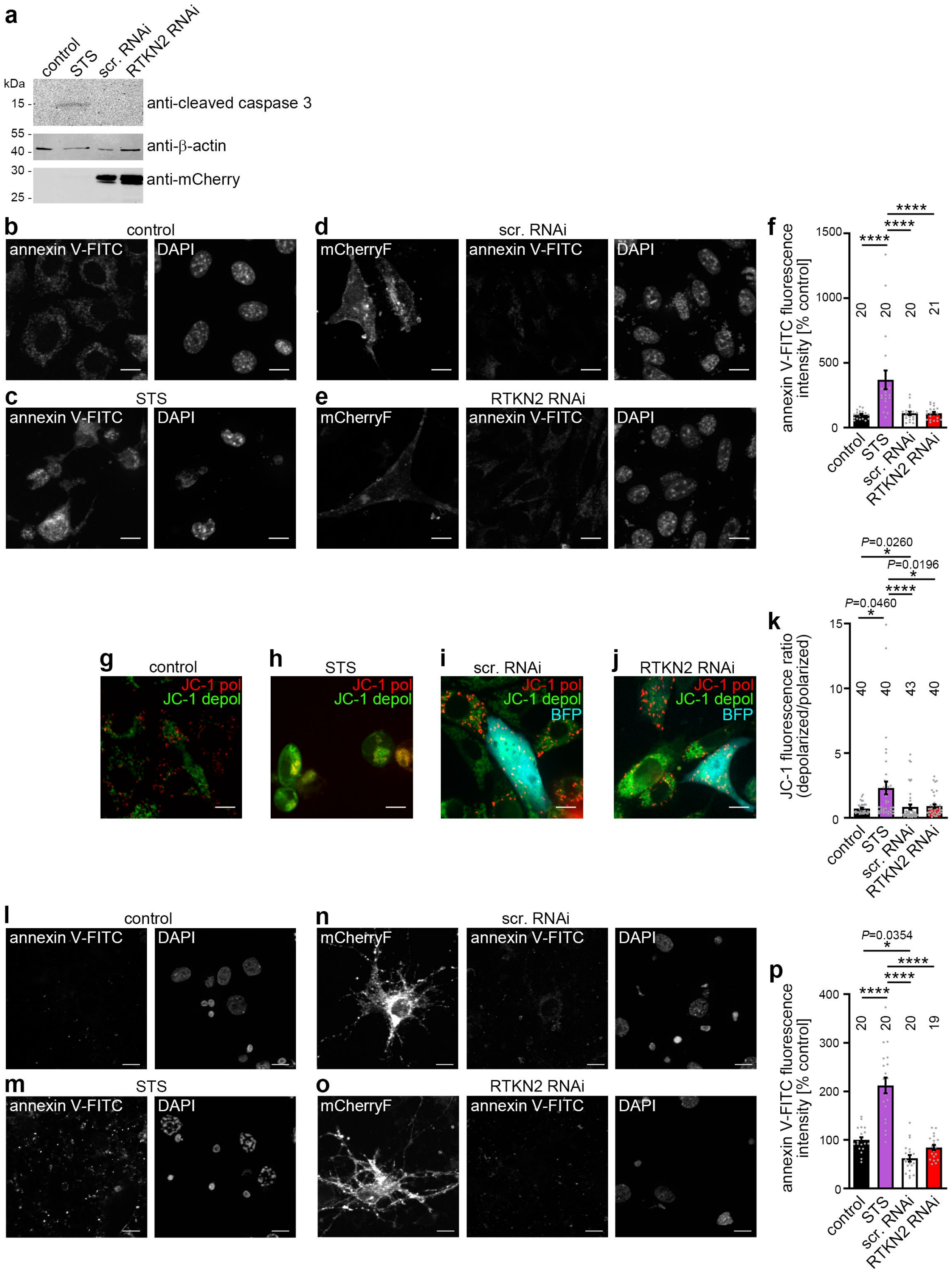
a
control
STS
scr. RNAi
RTKN2 RNAi
kDa
15 -
55 -
40 -
30 -
25 -
anti-cleaved caspase 3
anti-β-actin
anti-mCherry
b
control
annexin V-FITC
DAPI
c
STS
annexin V-FITC
DAPI
d
scr. RNAi
mCherryF
annexin V-FITC
DAPI
e
RTKN2 RNAi
mCherryF
annexin V-FITC
DAPI
f
annexin V-FITC fluorescence intensity [% control]
1500
1000
500
0
20
20
20
21
****
****
****
control
STS
scr. RNAi
RTKN2 RNAi
g
control
JC-1 pol
JC-1 depol
h
STS
JC-1 pol
JC-1 depol
i
scr. RNAi
JC-1 pol
JC-1 depol
BFP
j
RTKN2 RNAi
JC-1 pol
JC-1 depol
BFP
k
JC-1 fluorescence ratio (depolarized/polarized)
P=0.0260
P=0.0196
P=0.0460
*
*
*
****
15
10
5
0
40
40
43
40
control
STS
scr. RNAi
RTKN2 RNAi
l
control
annexin V-FITC
DAPI
m
STS
annexin V-FITC
DAPI
n
scr. RNAi
mCherryF
annexin V-FITC
DAPI
o
RTKN2 RNAi
mCherryF
annexin V-FITC
DAPI
p
annexin V-FITC fluorescence intensity [% control]
P=0.0354
*
****
****
****
400
300
200
100
0
20
20
20
19
control
STS
scr. RNAi
RTKN2 RNAi

Supplementary Fig. 10. Examinations of putative apoptotic effects of RNAi-mediated knockdown of rhotekin2 in both NIH3T3 cells and rat primary hippocampal neurons

(**a**) Immunoblotting analysis of activated, cleaved caspase 3 in control NIH3T3 cells, cells treated with staurosporine (STS; positive control) and cells expressing either scrambled or RTKN2 RNAi showed an activation of caspase 3 in STS-treated NIH3T3 cells but no effect upon RTKN2 RNAi. The experiment (shown as representative images) has been done at least twice with similar results. (**b-e**) Sum intensity projections of immunofluorescence images of control NIH3T3 cells (**b**), cells treated with STS (**c**) and cells transfected with either scrambled RNAi (**d**) or RTKN2 RNAi plasmids (**e**). Depicted are stainings with fluorescently labeled annexin V (annexin V-FITC) marking early apoptosis together with DAPI stainings marking the nuclei and the signal of farnesylated mCherry (mCherryF) coexpressed with scrambled RNAi (**d**) or RTKN2 RNAi (**e**) to identify transfected cells. (**f**) Quantification of annexin V-FITC intensity normalized to control NIH3T3 cells. (**g-j**) Sum intensity projections of immunofluorescence images of related experiments, in which JC-1 was used as a marker for mitochondrial polarization/depolarization. Transfected cells (**i,j**) were marked by expression of BFP (blue) as reporter. (**k**) Quantification of JC-1 intensity ratio of green versus red fluorescence. (**l-p**) Experiments addressing a putative apoptosis induction by rhotekin2-deficiency by annexin V-FITC labeling (as in **b-f**) using DIV4+2 primary rat hippocampal neurons. Data represent mean±SEM visualized as bar/dot plots. (**f**) n=20 (control, STS-treated, scr. RNAi), n=21 (RTKN2 RNAi) cells from 2 independent assays. (**k**) n=40 (control, STS-treated, RTKN2 RNAi), n=43 (scr. RNAi) cells from 3 independent assays. (**p**) n=20 (control, STS-treated, scr. RNAi), n=19 (RTKN2 RNAi) cells from 2 independent assays. Statistical analyses, one-way ANOVA and Tukey`s post-test. * P<0.05, **** P<0.0001 (for exact P values (≥0.0001) see figure).

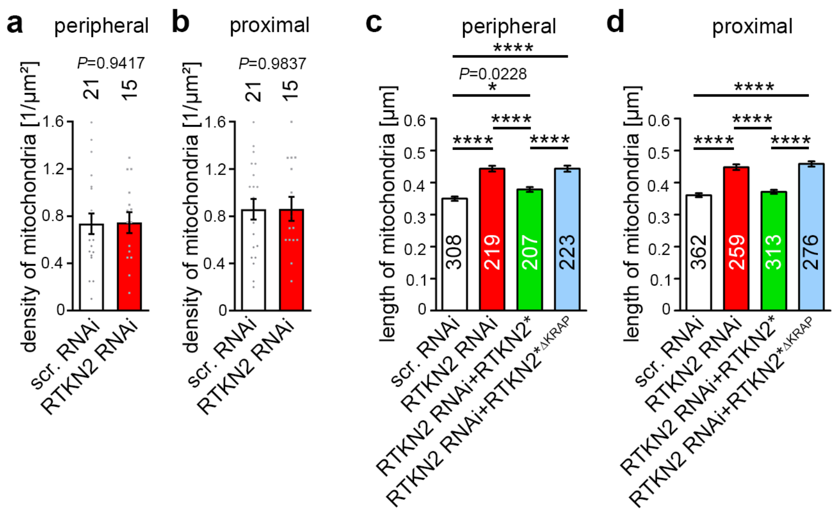

Supplementary Figure 11. Rhotekin2 deficiency did not change the distribution of mitochondria and caused mitochondrial length phenotypes that are similar in peripheral and proximal areas of the dendritic arbor

(**a,b**) Analysis of mitochondrial density within peripheral (**a**) and proximal (**b**) dendritic arbor segments of 20 µm in length each based on anti-cytochrome c-based detections of mitochondria. **(c,d)** Quantitative analyses of mitochondrial length in peripheral (**c**) and in proximal (**d**) dendritic segments of neurons demonstrating that the rhotekin2 loss-of-function effects, the successful rescue by reexpressing RNAi-insensitive rhotekin2 (RTKN2*) and the failed rescue attempt with RTKN2*$^{\Delta KRAP}$ were observed irrespective of the position of the mitochondria in the dendrites. Data, mean±SEM visualized as bar plots (**c,d**) and bar/dot plots for examinations with lower *n* numbers (**a,b**), respectively. (**a**) RNAi, *n*=21; RTKN2 RNAi, *n*=15 neurons; (**b**) RNAi, *n*=21; RTKN2 RNAi, n=15 neurons; (**c**) scr. RNAi, *n*=308; RTKN2 RNAi, *n*=219; RTKN2 RNAi+RTKN2*, *n*=207 and RTKN2 RNAi+RTKN2*$^{\Delta KRAP}$, *n*=223 mitochondria; (**d**) scr. RNAi, *n*=362; RTKN2 RNAi, *n*=259; RTKN2 RNAi+RTKN2*, *n*=313 and RTKN2 RNAi+RTKN2*$^{\Delta KRAP}$, *n*=276 mitochondria from 3 independent

assays. Statistical significances, Student´s unpaired t-test (**a,b**) and Kruskal-Wallis/Dunn´s post-test (**c,d**), respectively. * $P<0.05$, **** $P<0.0001$. For exact *P* values ≥ 0.0001 see figure.

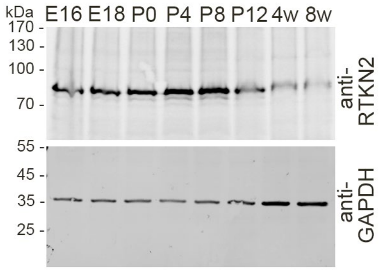

Supplementary Fig. 12. Rhotekin2 expression in the brain is high until about postnatal day 8 in mice and subsequently declines

Anti-rhotekin2 immunoblotting analyses of homogenates from embryonic (E16, E18) and postnatal (P0, P4, P8, P12, 4 weeks and 8 weeks) mouse brains. The experiment shown as representative images has been done twice with similar results. Note the strong rhotekin2 expression E16 to P8. Anti-GAPDH immunoblotting is shown for comparison.

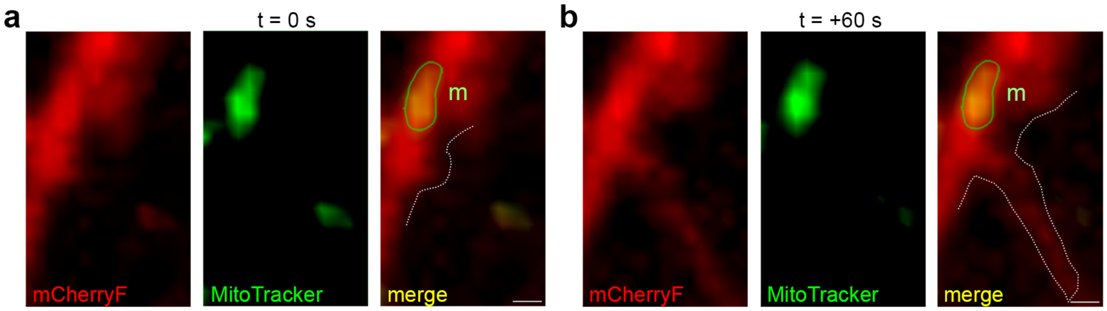

Supplementary Fig. 13. Mitochondria are aligned to a central position prior to branch induction

(**a,b**) Individual fluorescence channels of mCherryF (shown in red) and MitoTracker (shown in green) as well as the merged images of MIPs of the t = 0 s frame (**a**) and of the t = +60 s frame (**b**) of the 3D-time-lapse spinning disc microscopic recording of the dendritic branch induction event shown in **Fig. 4b** and **c**. Note that the mitochondrion (m) (outlined in green in the merge) is maintained at a position inside of a 1 µm wide dendritic segment representing the center of the dendritic branch induction zone (mCherryF-marked plasma membrane and its forming protrusion is outlined in white). Bars, 500 nm.

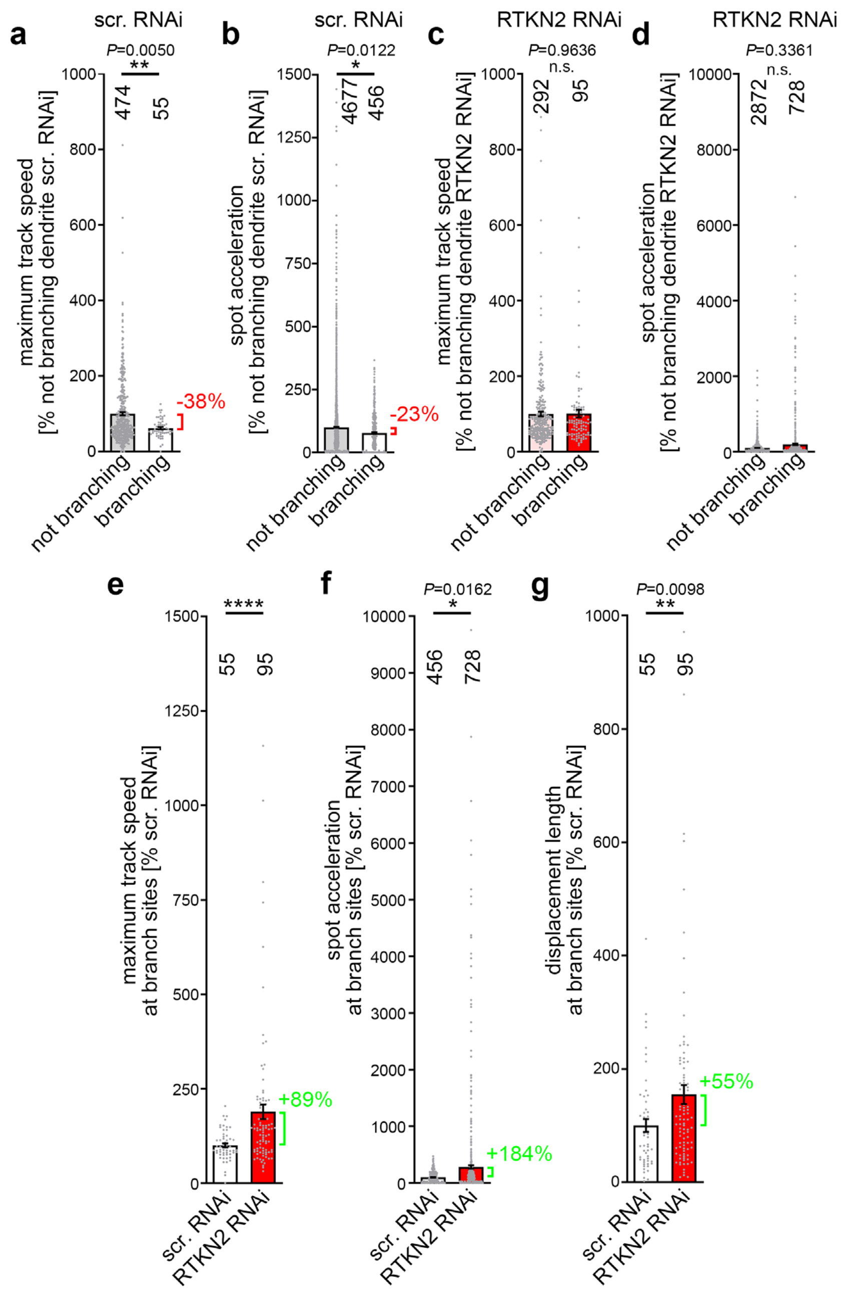

Supplementary Fig. 14. Rhotekin2-dependent attenuation of mitochondrial motility at dendritic branch sites as observed by imaging conditions providing a higher temporal resolution

(**a-d**) Quantitative analyses of 3D live examinations of dendritic mitochondria (marked by MitoTracker staining) at branching versus not branching sites in control DIV7 primary rat hippocampal neurons transfected with scr. RNAi (**a,b**) and neurons transfected with RTKN2 RNAi (**c,d**). Shown are

maximum track speeds (**a,c**) and spot accelerations (**b,d**). (**e-f**) Determinations of the mitochondrial motility parameters maximum track speed (**e**), spot acceleration (**f**) and displacement length (**g**) at branch induction sites in control (scr. RNAi) versus RTKN2 RNAi neurons. The examinations reflect those shown in **Fig. 6** but used an increased frame rate of only 2.4 s per frame at the expense of 3D resolution (only 3 z planes). Note that the data obtained by the imaging conditions with an increased temporal resolution are similar to those obtained by slower imaging but better 3D resolution (**Fig. 6**). Data, mean±SEM visualized as bar/dot plots due to in part lower *n* numbers. (**a**) Not branching, *n*=474; branching, *n*=55 maximum track speeds and (**b**) not branching, *n*=4677; branching, *n*=456 spot accelerations in control cells (scr. RNAi); (**c**) not branching, *n*=292 branching, *n*=95 maximum track speeds and (**d**) not branching, n=2872; branching, n=728 spot accelerations in RTKN2 RNAi neurons. (**e-g**) Scr. RNAi, *n*=55 and RTKN2 RNAi, *n*=95 maximum track speeds and displacement lengths, respectively, and scr. RNAi, *n*=456 and RTKN2 RNAi, *n*=728 spot accelerations. Statistical significances, Mann-Whitney. * $P<0.05$, ** $P<0.01$, **** $P<0.0001$. For exact *P* values ≥0.0001 see figure.

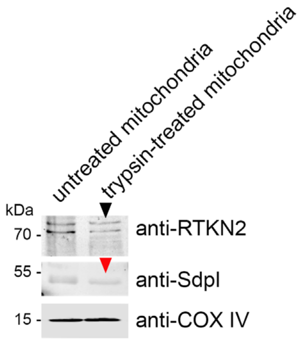

Supplementary Fig. 15. Trypsin-treatment of purified mitochondria leads to strong reductions of the amounts of endogenous rhotekin2 and syndapin I at mitochondria

Immunoblotting analyses of purified mitochondria from brain homogenates used for the reconstitution shown in **Fig. 6k.** Shown is the presence of endogenous rhotekin2 as well as of endogenous syndapin I (low amounts; high sensitivity scan) at the surfaces of equal amounts of purified mitochondria (untreated) and of trypsin-treated mitochondria. The experiment shown as representative images has been done at least twice with similar results. Note that the internal mitochondrial protein COX IV used as marker for the purified mitochondria was not affected by trypsin treatment, whereas immunostainings of proteins associated with the mitochondrial surface, such as rhotekin2 and syndapin I, showed reductions (black and red arrowhead, respectively).

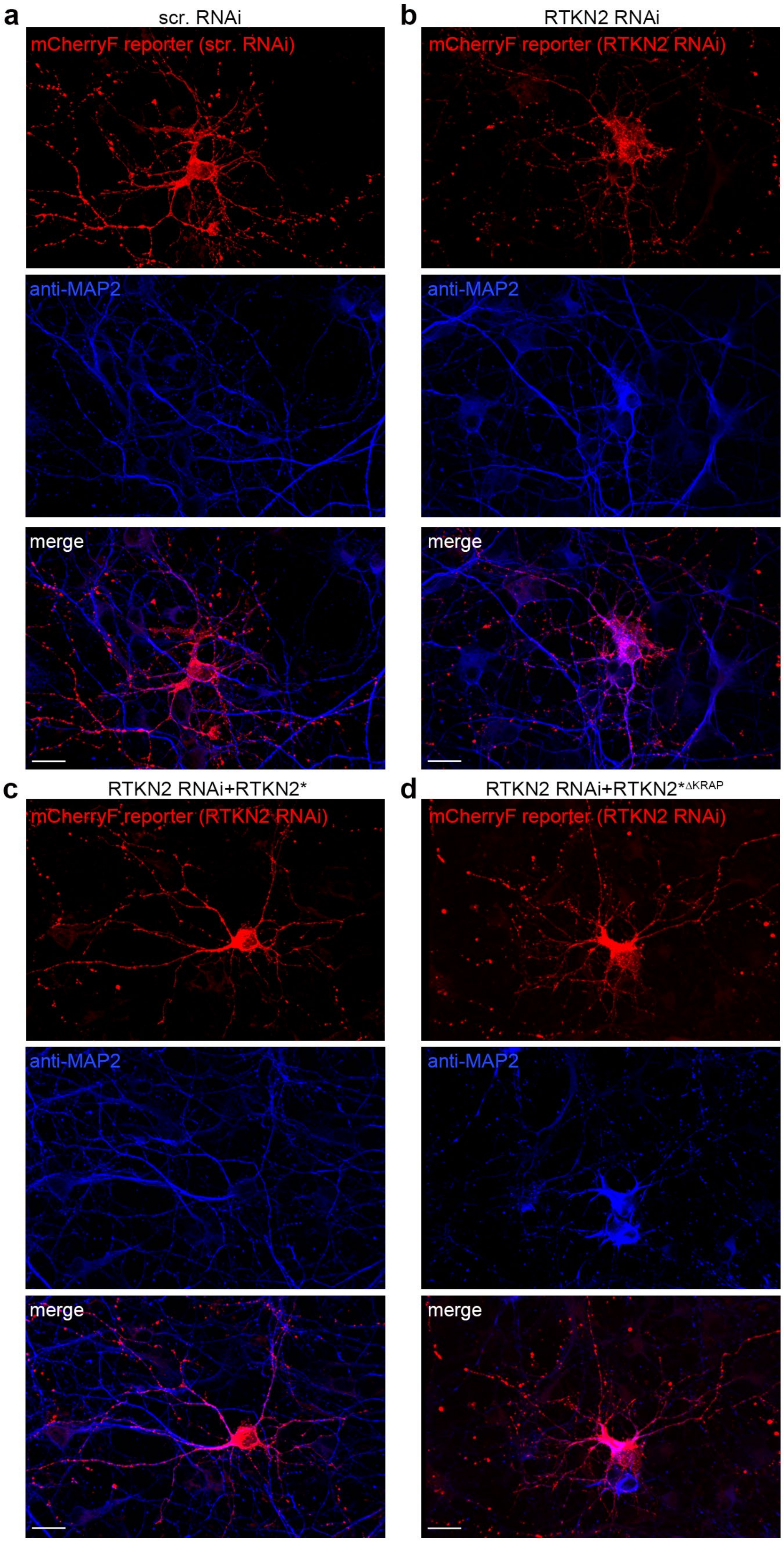
a
scr. RNAi
mCherryF reporter (scr. RNAi)
anti-MAP2
merge
b
RTKN2 RNAi
mCherryF reporter (RTKN2 RNAi)
anti-MAP2
merge
c
RTKN2 RNAi+RTKN2*
mCherryF reporter (RTKN2 RNAi)
anti-MAP2
merge
d
RTKN2 RNAi+RTKN2*ΔKRAP
mCherryF reporter (RTKN2 RNAi)
anti-MAP2
merge

Supplementary Fig. 16. The crucial mitochondria-positioning protein rhotekin2 is important for dendrite arbor formation in developing neurons

(**a-d**) Accompanying single channel images merged in **Fig. 8.** Shown are the anti-MAP2 immunostainings and the mCherryF signals (cell outlining; reporter for RNAi plasmids) as well as the merged images of the two channels of neurons transfected with scrambled RNAi control vector (**a**) and RTKN2 RNAi vectors **(b-d**) in absence (**b**) or in presence of full-length RNAi insensitive RTKN2* (**c**) or a rhotekin2 mutant lacking the syndapin I-binding KRAP motif (**d**). Untagged RTKN2* and RTKN2*$^{\Delta KRAP}$ were reported by GFP expression (not shown; see **Fig. 8**). Bars, 10 μm.

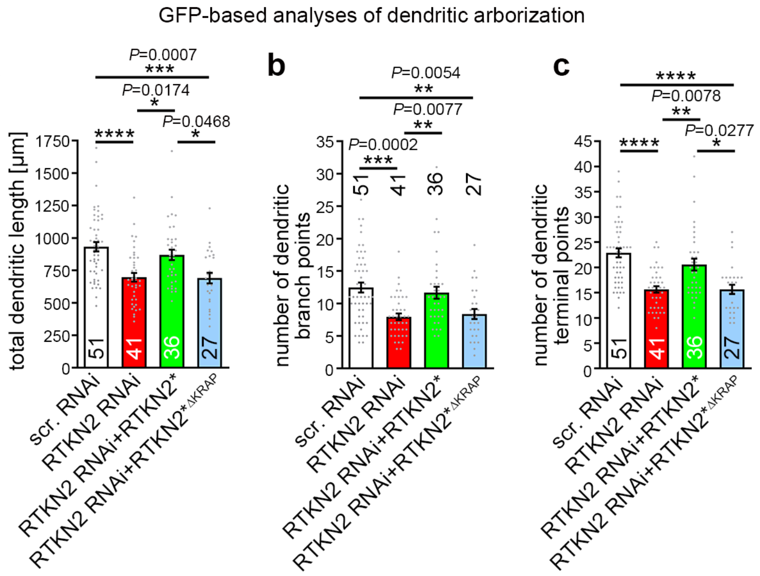

Supplementary Fig. 17. The crucial mitochondria-positioning protein rhotekin2 is important for a proper dendritic arbor in developing neurons

(**a-c**) Analyses of dendritic rhotekin2 loss-of-function phenotypes and rescue attempts in primary hippocampal neurons transfected at DIV4, fixed and immunostained for MAP2 48 h thereafter and subjected to GFP-based analyses of neuronal morphology by 3D-surface reconstructions and morphology tracking using Imaris software. Confirmations of the dendritic nature of GFP-tracked protrusions were done based on anti-MAP2 colabeling. Neurons were transfected with either scrambled RNAi control vector (scr. RNAi) or with RTKN2 RNAi vectors (all mCherryF-reported) in absence (RTKN2 RNAi) (**b**) or in presence of full-length RNAi insensitive rhotekin2 (RTKN2 RNAi+RTKN2*) (**c**) or a rhotekin2 mutant lacking the syndapin I-binding KRAP motif (RTKN2 RNAi+RTKN2*$^{\Delta KRAP}$) (**d**), marked by coexpression of the reporter and cell filler GFP. Shown are quantitative determinations of the total dendritic length (**a**), number of dendritic branch points (**b**) and number of dendritic terminal points (**c**). Note that the results do not differ from the anti-MAP2-based analyses shown in **Figure 8**. (**a-**

c) *n*=51 (scr. RNAi), *n*=41 (RTKN2 RNAi), *n*=36 (RTKN2 RNAi+RTKN2*), *n*=27 (RTKN2 RNAi+RTKN2*$^{\Delta KRAP}$) neurons for each condition (from 3 independent assays). Data, mean±SEM visualized as bar/dot plots. Statistical significances, Kruskal-Wallis/Dunn's. * $P<0.05$; ** $P<0.01$; *** $P<0.001$; **** $P<0.0001$. For exact *P* values ≥0.0001 see figure.
