# Supplementary material for: Mitochondria are positioned at dendritic branch induction sites, a process requiring rhotekin2 and syndapin I
Source: Nat Commun. 2025 Mar 10;16:2353. doi: 10.1038/s41467-025-57399-0 (PMC11893792; doi:10.1038/s41467-025-57399-0)
Supplement: Supplementary file 2 — Description of Additional Supplementary Files [file 41467_2025_57399_MOESM2_ESM.pdf]

Description of Additional Supplementary Files:

Supplementary Movie 1: Imaris-animated example video (shown as 24 frames/s video with key frames corresponding to those recorded by 3D-spinning disc imaging) of a developing primary hippocampal neuron at DIV7 that was transfected with scr. RNAi and with mCherryF (tracer of the plasma membrane; red) at DIV4 and stained with MitoTracker to trace mitochondria (white). For the analyzed time frame from -60 s to +60 s, mitochondria are additionally shown as spot reconstruction and with their corresponding trajectories. Two additional elements were added for illustration of the example video. Element 1 (white line) marks the 3 μm-zone of analysis composed of the central area of dendritic branch induction and the neighboring distal and proximal areas. Element 2 (white dot above dendrite) marks the site of dendritic protrusion induction at t = 0 s.

Supplementary Movie 2: Imaris-animated example video (shown as 24 frames/s video with key frames corresponding to those recorded by 3D-spinning disc imaging) of a developing primary hippocampal neuron at DIV7 that was transfected with rhotekin2 RNAi and with mCherryF (tracer of the plasma membrane; red) at DIV4 and stained with MitoTracker to trace mitochondria (white). For the analyzed time frame from -60 s to +60 s, mitochondria are additionally shown as spot reconstruction and with their corresponding trajectories. Two additional elements were added for illustration of the example video. Element 1 (white line) marks one example of a 3 μm-zone of analysis composed of the central area of dendritic branch induction and the neighboring distal and proximal areas. Element 2 (white dot above dendrite) marks the site of dendritic protrusion induction at t = 0 s. Note that an additional dendritic branch initiation event (not marked; also without mitochondrion at central position) occurs at the left side of the area shown (protruding downwards).

Supplementary Movie 3: Imaris-animated example video (shown as 24 frames/s video with key frames corresponding to those recorded by 3D-spinning disc imaging) of a developing primary hippocampal neuron at DIV7 that was transfected with scr. RNAi and with mCherryF (tracer of the plasma membrane; red) at DIV4 and stained with MitoTracker to trace mitochondria (white). For the analyzed time frame from -60 s to +60 s, mitochondria are additionally shown as spot reconstruction and with their corresponding trajectories. Two additional elements were added for illustration of the example video. Element 1 (white line) marks the 3 µm-zone of analysis composed of the central area of dendritic branch induction and the neighboring distal and proximal areas. Element 2 (white dot above dendrite) marks the site of dendritic protrusion induction at t = 0 s.

Supplementary Movie 4: Imaris-animated example video (shown as 24 frames/s video with key frames corresponding to those recorded by 3D-spinning disc imaging) of a developing primary hippocampal neuron at DIV7 that was transfected with syndapin I RNAi and with mCherryF (tracer of the plasma membrane; red) at DIV4 and stained with MitoTracker to trace mitochondria (white). For the analyzed time frame from -60 s to +60 s, mitochondria are additionally shown as spot reconstruction and with their corresponding trajectories. Two additional elements were added for illustration of the example video. Element 1 (white line) marks the 3 µm-zone of analysis composed of the central area of dendritic branch induction and the neighboring distal and proximal areas. Element 2 (white dot above dendrite) marks the site of dendritic protrusion induction at t = 0 s.
