# Supplementary figures and images for: Mitochondria are positioned at dendritic branch induction sites, a process requiring rhotekin2 and syndapin I
Source: Nat Commun. 2025 Mar 10;16:2353. doi: 10.1038/s41467-025-57399-0 (PMC11893792; doi:10.1038/s41467-025-57399-0)

# Source Data Figure 1

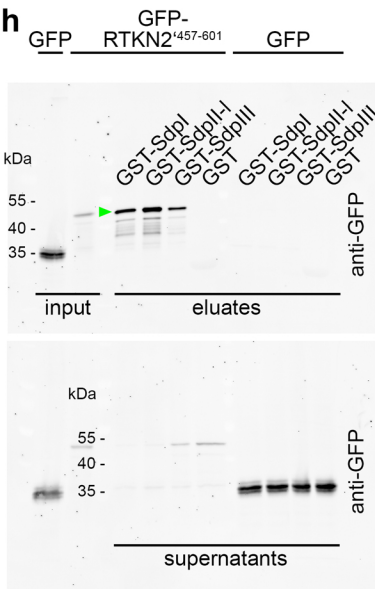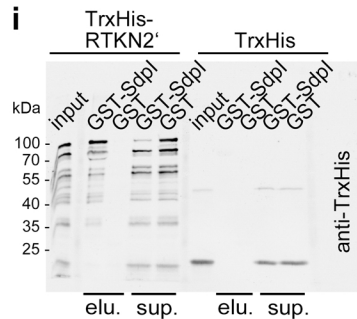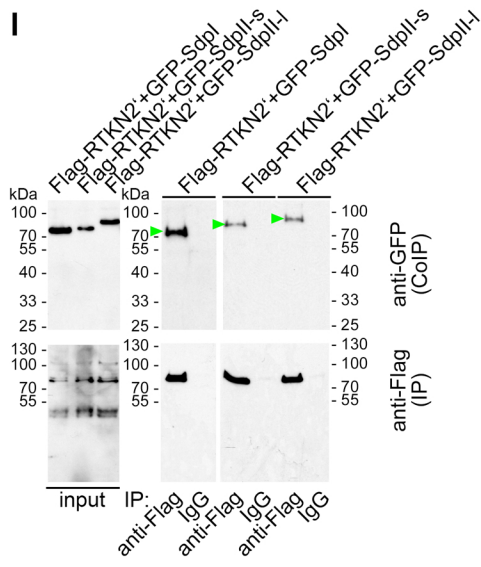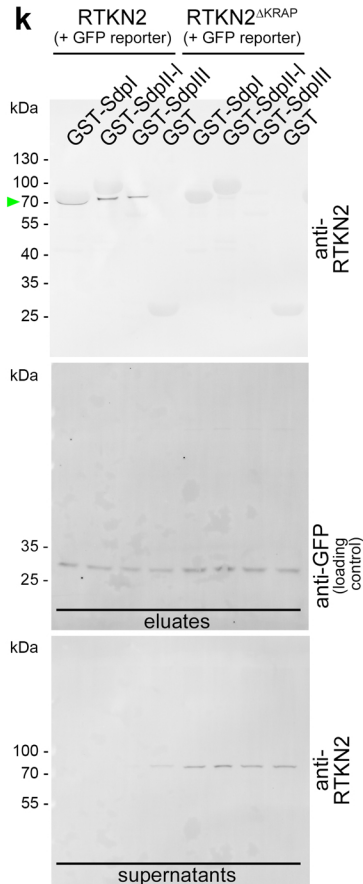

Supplement: Supplementary file 9 — Source Data [file 41467_2025_57399_MOESM9_ESM.zip › Kessels/482576_3_data_set_10322491_srnmyy.pdf]

Source Data Figure 2

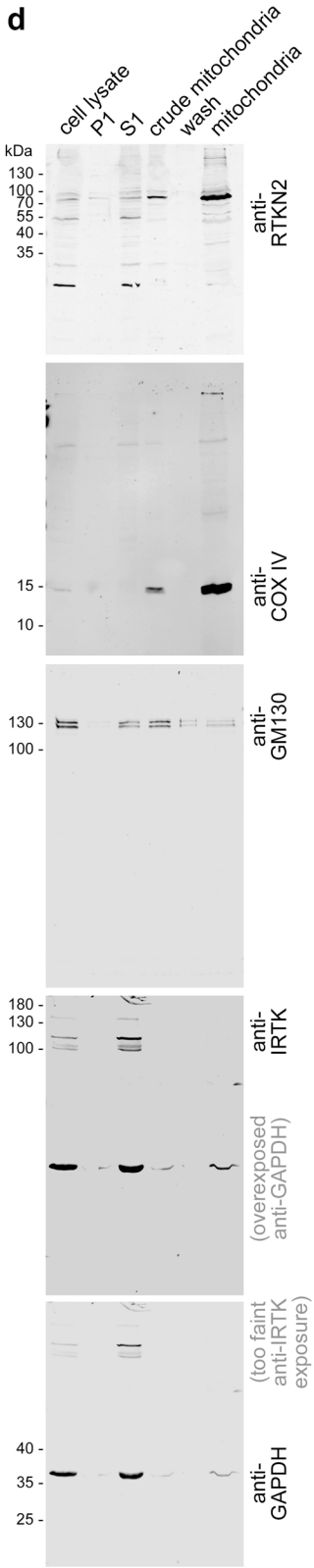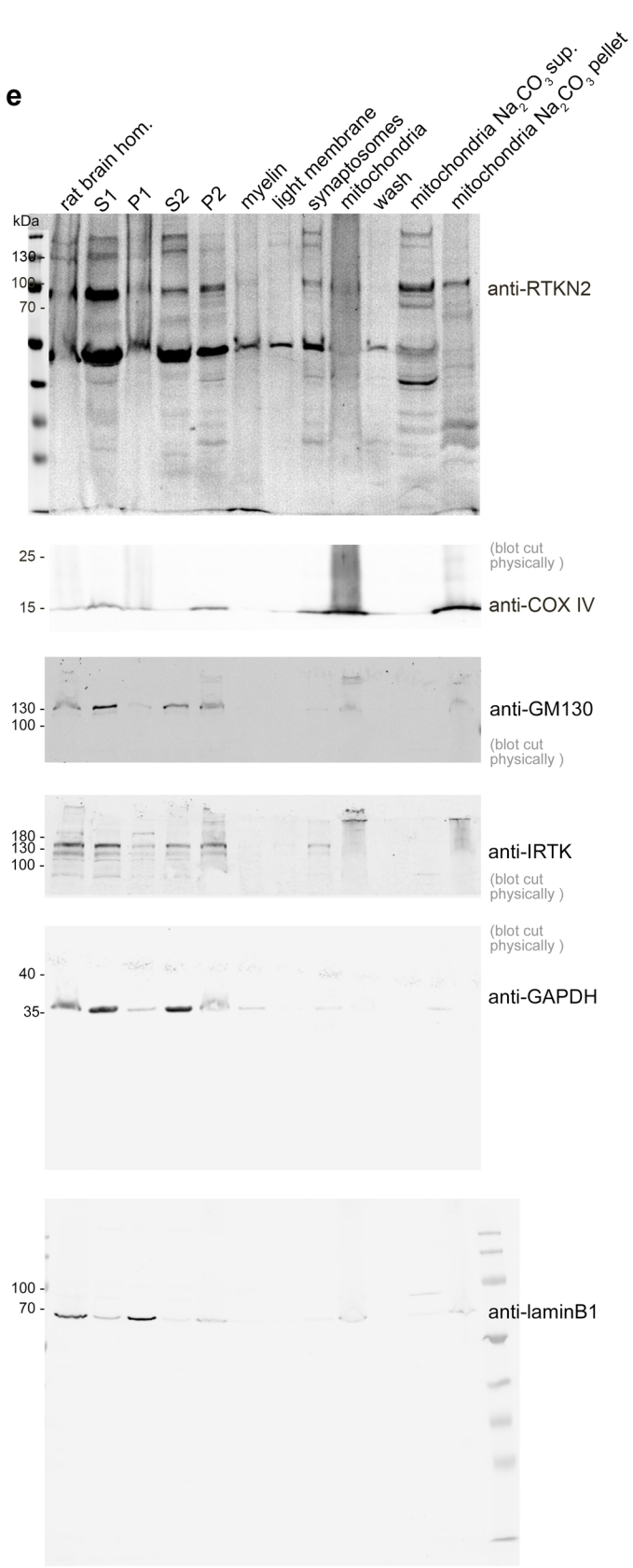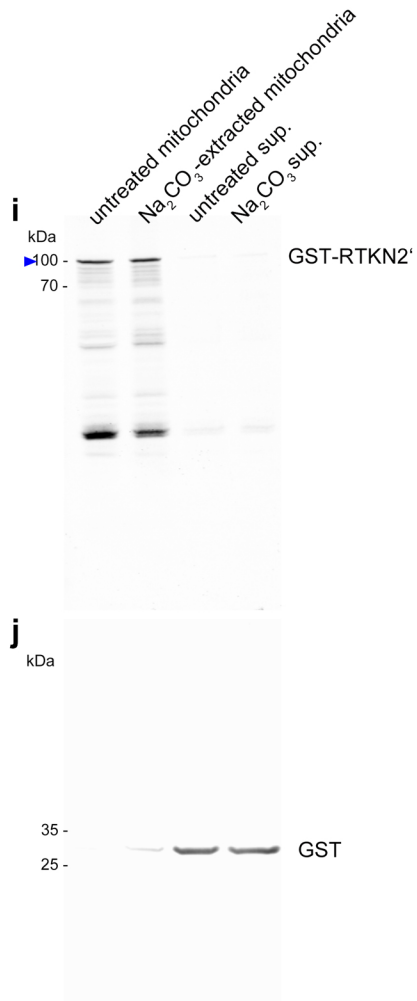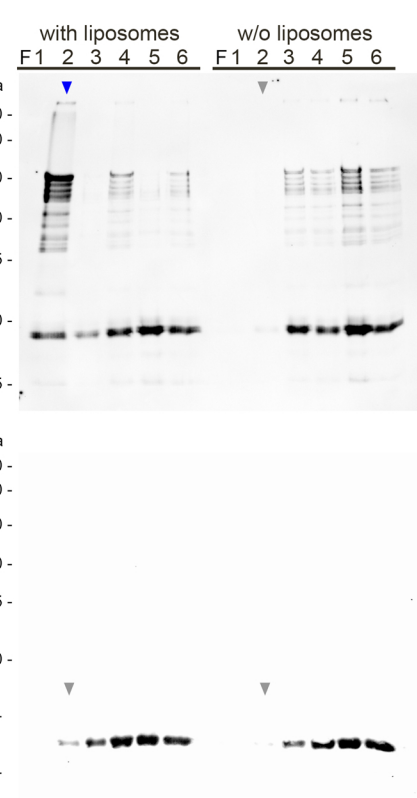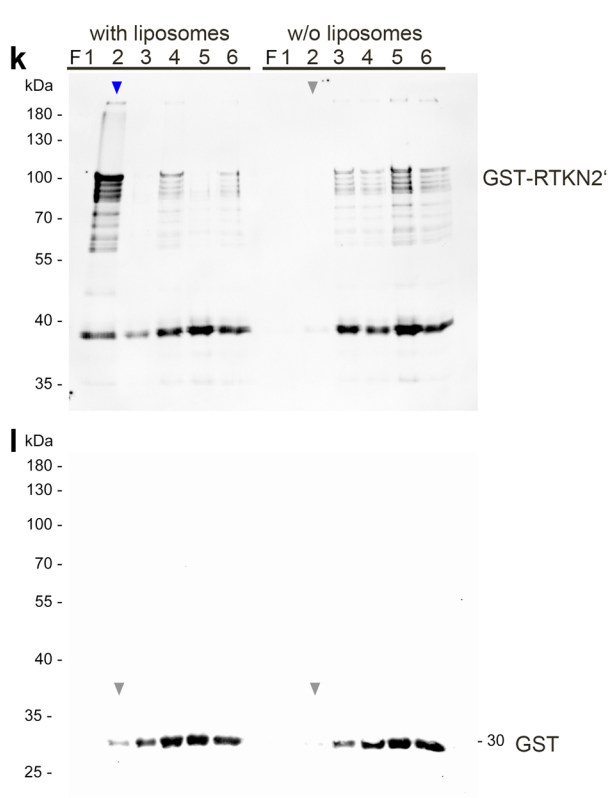

Supplement: Supplementary file 9 — Source Data [file 41467_2025_57399_MOESM9_ESM.zip › Kessels/482576_3_data_set_10322492_sr9myy.pdf]

# Source Data Figure 3

**f**

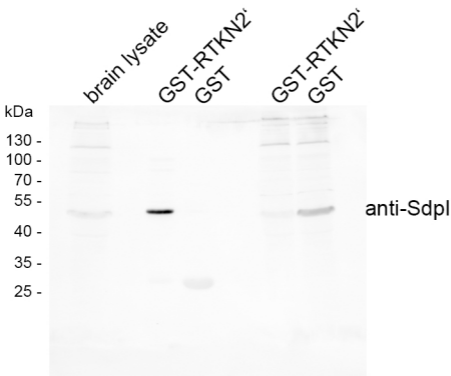

**g**

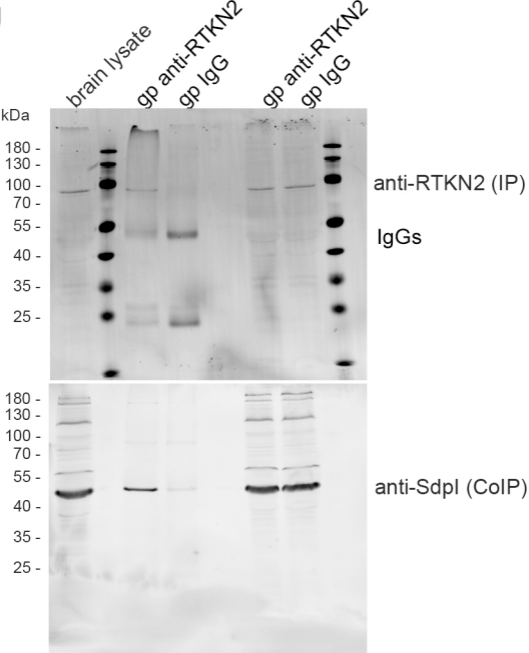

Supplement: Supplementary file 9 — Source Data [file 41467_2025_57399_MOESM9_ESM.zip › Kessels/482576_3_data_set_10322493_sr4n1b.pdf]

# Source Data Figure 6

k

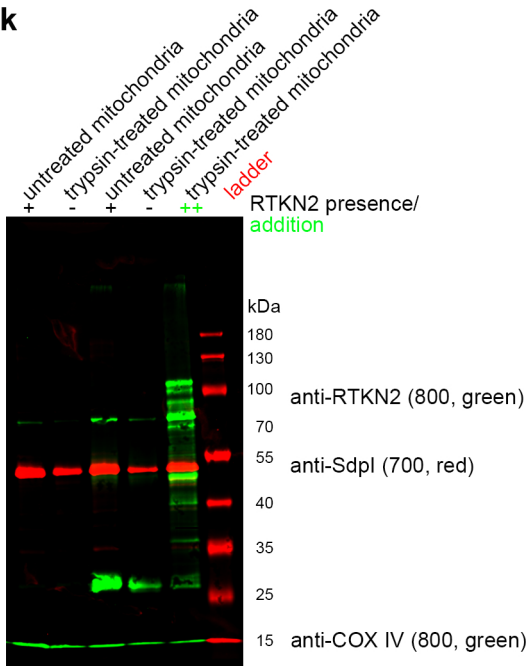

Supplement: Supplementary file 9 — Source Data [file 41467_2025_57399_MOESM9_ESM.zip › Kessels/482576_3_data_set_10322494_sr8myy.pdf]

# Source Data Supplementary Figure 4

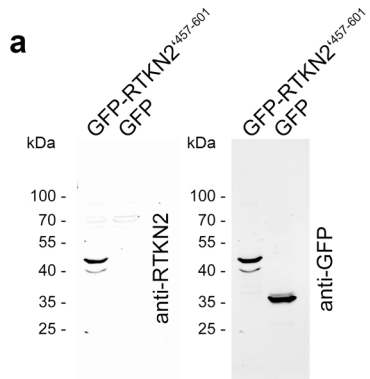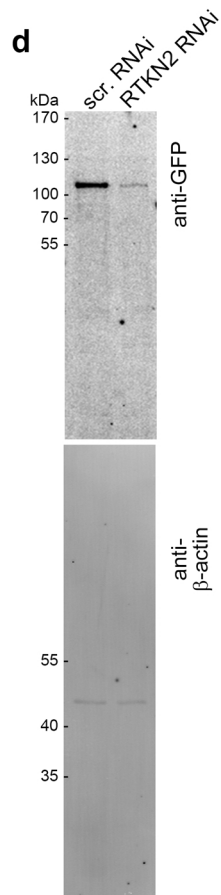

Supplement: Supplementary file 9 — Source Data [file 41467_2025_57399_MOESM9_ESM.zip › Kessels/482576_3_data_set_10322495_srwmyy.pdf]

# Source Data Supplementary Figure 9

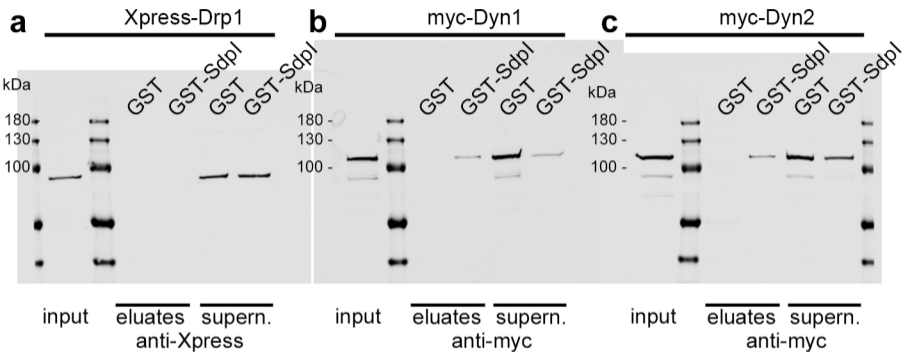

Supplement: Supplementary file 9 — Source Data [file 41467_2025_57399_MOESM9_ESM.zip › Kessels/482576_3_data_set_10322496_srpmyy.pdf]

# Source Data Supplementary Fig. 10

**a**

control  
STS  
scr. RNAi  
RTKN2 RNAi

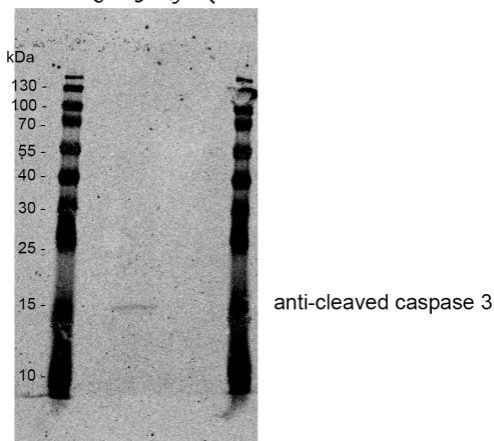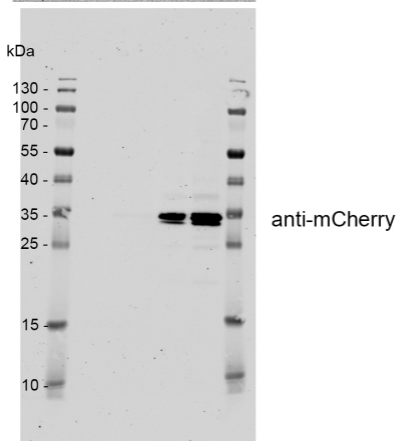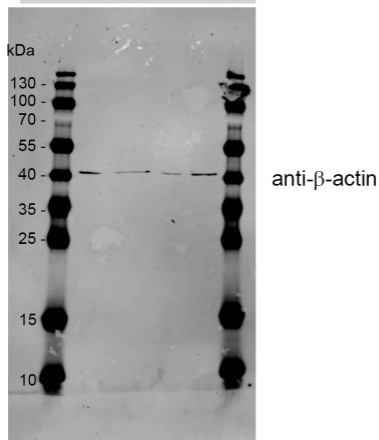

Supplement: Supplementary file 9 — Source Data [file 41467_2025_57399_MOESM9_ESM.zip › Kessels/482576_3_data_set_10322497_srymyz.pdf]

# Source data Suppl. Fig. 12

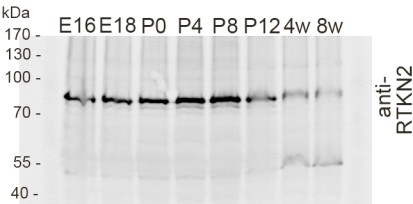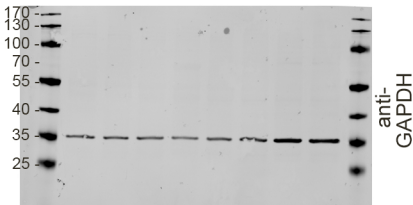

Supplement: Supplementary file 9 — Source Data [file 41467_2025_57399_MOESM9_ESM.zip › Kessels/482576_3_data_set_10322498_srjmyz.pdf]

# Source Data Supplementary Fig. 15

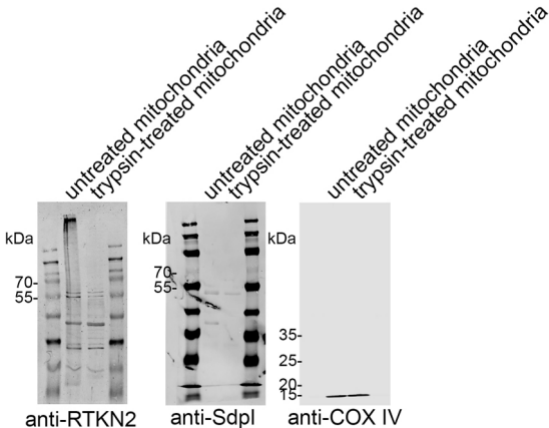

Supplement: Supplementary file 9 — Source Data [file 41467_2025_57399_MOESM9_ESM.zip › Kessels/482576_3_data_set_10322499_srlmyz.pdf]
